# A Multi-Sensor Machine Learning Framework Integrating UAV Multispectral Imagery and LiDAR Data for Living Biomass Carbon Stock Estimation in Silviculturally Treated Forests

**DOI:** 10.3390/s26144496

**Published:** 2026-07-15

**Authors:** Nyo Me Htun, Toshiaki Owari, Satoshi N. Suzuki, Songqiu Deng, Tetsuyuki Kobayashi, Sakura Asato, Akio Oshima, Mutsuki Hirama, Koichi Takahashi, Yasuo Isozaki, Takumi Okahira, Satoshi Kita, Ryota Konda, Manato Fushimi

**Affiliations:** 1 The University of Tokyo Hokkaido Forest, Graduate School of Agricultural and Life Sciences, The University of Tokyo, Furano 079-1563, Hokkaido, Japan; owari@g.ecc.u-tokyo.ac.jp (T.O.); deng@uf.a.u-tokyo.ac.jp (S.D.); k-tetsuyuki@uf.a.u-tokyo.ac.jp (T.K.); asato@uf.a.u-tokyo.ac.jp (S.A.); a-ooshima@g.ecc.u-tokyo.ac.jp (A.O.); hirama@uf.a.u-tokyo.ac.jp (M.H.); takakou@uf.a.u-tokyo.ac.jp (K.T.); yisozaki@uf.a.u-tokyo.ac.jp (Y.I.); okahira@uf.a.u-tokyo.ac.jp (T.O.); 2 Nakagawa Experimental Forest, Field Science Center for Northern Biosphere, Hokkaido University, Otoineppu 098-2501, Hokkaido, Japan; snsuzuki@fsc.hokudai.ac.jp; 3 Forest and Landscape Research Center, Sumitomo Forestry Co., Ltd., Chiyoda 100-8270, Tokyo, Japan; kita_satoshi@star.sfc.co.jp; 4 Tsukuba Research Institute, Sumitomo Forestry Co., Ltd., Tsukuba 300-2646, Ibaraki, Japan; konda_ryouta@star.sfc.co.jp (R.K.); fushimi_manato@star.sfc.co.jp (M.F.)

**Keywords:** multi-sensor, machine learning, carbon stock, silviculturally treated forests

## Abstract

The accurate and scalable estimation of carbon stocks in living biomass remains challenging in structurally heterogeneous forests subjected to different silvicultural treatments. This study presents a multi-sensor machine learning framework that integrates unmanned aerial vehicle (UAV)-derived multispectral imagery with UAV- and airborne light detection and ranging (LiDAR) data for spatially explicit carbon stock estimation in managed forests of central and eastern Hokkaido, northern Japan. Field measurements from 38 plots were used for model development and validation. Spectral features derived from UAV multispectral imagery and structural metrics derived from UAV and airborne LiDAR data were integrated within a multi-sensor framework and evaluated using Multiple Linear Regression (MLR), Random Forest (RF), and Extreme Gradient Boosting (XGBoost), with MLR serving as a baseline model. A key objective was to quantify the relative contributions of spectral and structural sensing information for carbon stock estimation in silviculturally treated forests through the systematic comparison of canopy height model (CHM)-only, RGB + CHM, and multispectral + CHM datasets. The machine learning models consistently outperformed the baseline MLR model, with XGBoost generally outperforming RF and achieving a maximum validation R^2^ of 0.88 and root mean squared error (RMSE) of 27.41 Mg C ha^−1^. Although the improvement in plot-level prediction accuracy over the CHM-only configuration was modest, integrating multispectral imagery with LiDAR-derived structural metrics reduced prediction errors and systematic bias in wall-to-wall carbon stock mapping. These findings highlight the complementary roles of structural and spectral remote sensing information for spatially explicit carbon stock estimation in silviculturally treated forests.

## 1. Introduction

Forest ecosystems play a crucial role in sequestering atmospheric carbon dioxide, and the reliable quantification of carbon stored in living biomass is essential for understanding terrestrial carbon dynamics, supporting climate change mitigation strategies [1,2], forest management planning [3], and the evaluation of climate-smart silvicultural practices [4,5]. However, achieving accurate and scalable living biomass carbon stock estimation remains challenging across diverse forest management contexts, including plantations, scarified and site-prepared areas, and managed natural forests. These systems exhibit substantial variability in stand structure and species composition due to silvicultural interventions—such as thinning, clear-cutting, scarification, enrichment planting, and tending—implemented at different intensities and times. This management-driven heterogeneity modifies tree size distributions, canopy complexity, understory development, and biomass allocation patterns [6,7], ultimately leading to variation in carbon storage.

Traditional field-based forest inventories provide reliable measurements of tree-level attributes such as diameter at breast height (DBH) and height, from which biomass and carbon stocks can be derived using allometric equations [8,9,10]. Despite their considerable accuracy, these methods are labor-intensive, time-consuming, and spatially limited, making them difficult to upscale to landscape or regional scales [11,12,13]. In managed forests, this challenge is further exacerbated by the spatial heterogeneity introduced by different silvicultural treatments, canopy structural conditions, and disturbance histories, which reduce the representativeness of sparse field plots and increase uncertainty when extrapolating carbon estimates beyond sampled areas [14,15]. Consequently, there is an increasing demand for operationally scalable approaches capable of characterizing fine-scale spatial variability in carbon stocks across heterogeneously managed forest landscapes.

Remote sensing has emerged as a powerful tool for overcoming these limitations by providing spatially continuous observations of forest characteristics [16,17,18]. In particular, unmanned aerial vehicle (UAV)-based multispectral imagery offers a very high spatial resolution and flexible data acquisition, enabling the detailed characterization of the forest canopy’s spectral properties. Spectral indices derived from UAV multispectral data, such as the Normalized Difference Vegetation Index (NDVI), Green NDVI (GNDVI), and Normalized Difference Red Edge (NDRE), have been widely used as proxies for vegetation vigor, photosynthetic activity and variability in aboveground carbon stocks [19,20,21]. However, spectral information alone is often insufficient for accurately estimating forest biomass and carbon stocks, because it provides a limited representation of vertical canopy structure and tree size, especially in structurally complex or managed forests [21,22]. In addition, UAV-based observations are inherently sensitive to acquisition conditions, illumination variability, and site-specific characteristics, which may influence the prediction consistency and limit reproducibility across studies and forest environments.

Light detection and ranging (LiDAR) technology addresses this limitation by directly capturing three-dimensional forest structure. LiDAR-derived metrics, including canopy height, height percentiles, and canopy cover, have demonstrated strong relationships with aboveground biomass and carbon stocks across a range of forest types [21,23,24,25]. Airborne LiDAR has been widely applied in forest inventory and biomass estimation [24,26,27], but its high operational costs and limited temporal availability can restrict repeated or fine-scale assessments [28]. Furthermore, recent advances in UAV-based LiDAR systems have expanded their application in forest resource monitoring [22,29,30]. Although multi-sensor data fusion has consistently demonstrated higher predictive accuracy than single-sensor approaches [31,32,33], the effective integration of data from multiple sensors—such as high-resolution UAV multispectral imagery and airborne and UAV-based LiDAR—for forest carbon stock estimation remains underexplored, particularly in forests subject to active management. Combining spectral and structural information from UAV multispectral imagery and LiDAR has the potential to improve forest carbon stock estimation by capturing both canopy physiological properties and three-dimensional structure.

In recent years, numerous studies have successfully demonstrated the effectiveness of machine learning approaches for forest parameter estimation and carbon stock assessment [11,13,34,35,36]. These methods enable accurate predictions within a practical time frame while requiring relatively affordable computational resources, making them suitable for large-area forest monitoring. However, different machine learning algorithms often yield varying levels of accuracy even when applied to the same dataset, due to differences in model structure and learning mechanisms [37,38]. Among the available approaches, ensemble-based methods such as Random Forest (RF), and Extreme Gradient Boosting (XGBoost) have been widely adopted in forest carbon stock estimations [13,24,35,36,38,39]. These algorithms are particularly well suited for modeling complex systems due to their ability to capture nonlinear relationships and interactions among predictor variables [11,35,38], which makes them advantageous for integrating multi-source remote sensing data, including spectral indices and structural metrics.

However, their application using spectral information derived from satellite imagery is often constrained by the spatial resolution [40], especially in heterogeneous or silviculturally treated forests, where the canopy structure and disturbance intensity vary substantially over short spatial distances. High-resolution UAV imagery and LiDAR data provide greater capacity to capture fine-scale spatial heterogeneity associated with silvicultural treatments, thereby supporting more spatially explicit carbon stock estimation. Many studies have primarily employed machine learning approaches using UAV-derived RGB imagery combined with canopy height model (CHM) metrics or LiDAR-derived structural metrics alone for forest carbon stock estimation [24,25,41,42,43]. Although these approaches have shown promising results, they may provide limited representation of treatment-induced variability in heterogeneous managed forests and may reduce applicability for operational forest management.

Despite recent advances in remote sensing-based carbon mapping, relatively few studies have systematically compared progressively more informative sensing configurations—LiDAR-derived CHM only, UAV-derived RGB imagery + LiDAR-derived CHM, and UAV-derived multispectral imagery + LiDAR-derived CHM—to quantify the incremental contribution of spectral information to spatially explicit forest carbon stock estimation in forests subjected to contrasting silvicultural treatments. Existing studies have often focused on natural or unmanaged forests, relatively simple urban forests, species-specific analyses, or plot-level biomass and carbon stock estimation, limiting their applicability to operational carbon stock mapping in heterogeneous managed forests [21,44,45].

To address this gap, this study develops a spatially explicit multi-sensor machine learning framework for wall-to-wall carbon stock estimation, enabling continuous carbon stock predictions across the entire study area, in silviculturally treated forests of Hokkaido, Japan. In this study, silviculturally treated forests refer to forest stands that have been subjected to different management interventions, including plantation establishment, scarification treatments following typhoon disturbance with associated control areas, and managed mixed forests with varying thinning intensities (0%, 15%, and 35% cutting rates). These treatments have resulted in substantial variation in stand structure, species composition, and biomass accumulation, providing an ideal setting for evaluating the contribution of structural (CHM) and multispectral information to carbon stock estimation.

Building upon this, the primary contribution of this study is the systematic evaluation of different sensing configurations for forest carbon stock estimation using a consistent multi-sensor machine learning framework. By systematically comparing CHM-only, RGB + CHM, and multispectral + CHM sensing configurations, this study quantifies the additional predictive value of spectral information beyond LiDAR-derived structural information and identifies the sensing configuration that achieves the highest prediction accuracy across forests subjected to contrasting silvicultural treatments. The proposed machine learning framework is further benchmarked against a conventional Multiple Linear Regression (MLR) model to evaluate the added value of nonlinear machine learning approaches. The optimal sensing configuration and corresponding machine learning model are further applied to produce wall-to-wall carbon stock maps and evaluate their potential for operational carbon monitoring in structurally heterogeneous managed forests.

Accordingly, the objectives of this study are to: (1) quantify the incremental predictive value of adding spectral information to LiDAR-derived structural information for forest carbon stock estimation through systematic comparison of CHM-only, RGB + CHM, and multispectral + CHM sensing configurations; (2) compare the performance of XGBoost and RF models while benchmarking them against a baseline MLR model across the evaluated sensing configurations; and (3) apply the optimal sensing configuration and corresponding machine learning model to produce wall-to-wall carbon stock maps and assess their potential for operational carbon monitoring in structurally heterogeneous managed forests.

## 2. Materials and Methods

### 2.1. Study Site

**Figure 1:**
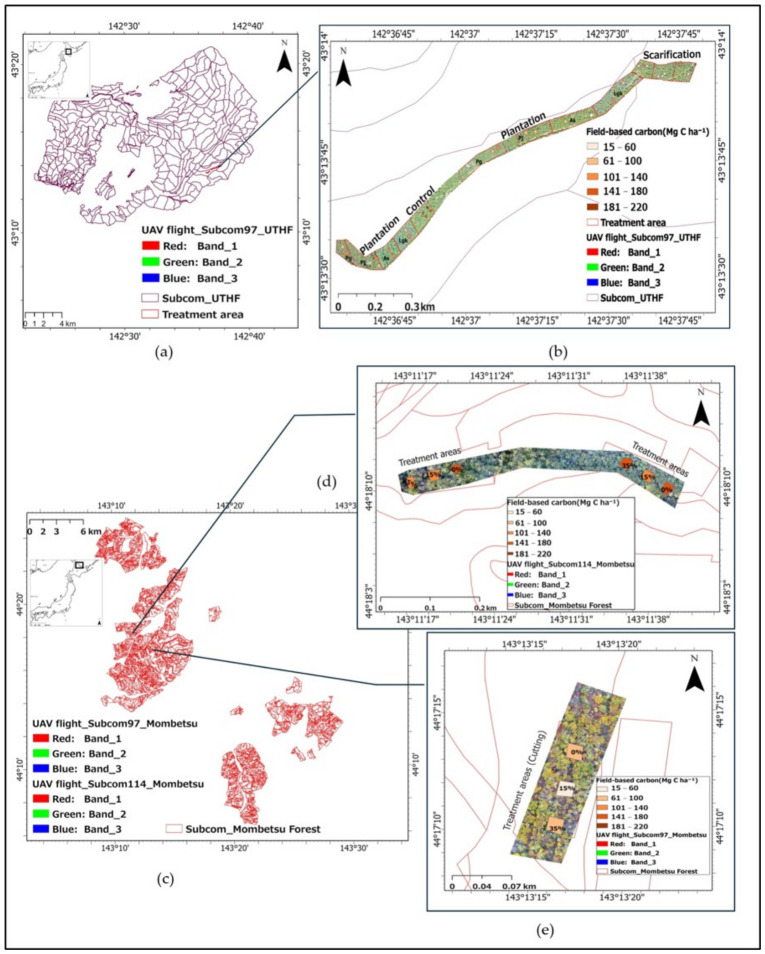
(**a**) Location map of the University of Tokyo Hokkaido Forest in central Hokkaido, Japan; (**b**) the UAV flight area in Sub-Compartment 97BC; (**c**) location map of Mombetsu forest in eastern part of Hokkaido, Japan; (**d**,**e**) the UAV flight areas in Sub-Compartments 114v&w and 97g of Mombetsu forest.

This study was conducted in four forest sub-compartments representing different disturbance and management conditions. One sub-compartment is situated in the University of Tokyo Hokkaido Forest (UTHF), central Hokkaido (43°10′–20′ N, 142°18′–40′ E) (Figure 1a,b), and was heavily affected by a severe typhoon in 1981 [46]. The remaining three sub-compartments are located in a managed forest holding of Sumitomo Forestry Co., Ltd. (hereafter referred to as Mombetsu forest), in Mombetsu City, eastern Hokkaido, Japan (44°07′–32′ N, 142°54′–29′ E) (Figure 1c–e) and were all subjected to the same silvicultural treatment, namely partial cutting, although the forest types differed (e.g., tall broadleaf dominated and short broadleaf dominated mixed forests).

In the UTHF, the study was carried out within Sub-Compartment 97BC (hereafter referred to as Sub-Compartment 97BC). The study area occupies a ridge characterized by flat to gently inclined terrain (<15%) at an elevation of approximately 580–670 m above sea level (a.s.l.). It experiences a cool–temperate climatic regime. According to meteorological records from the arboretum station of the UTHF located at 230 m a.s.l., the mean annual temperature was 6.6 °C and the annual precipitation totaled 1196 mm during the period 2011–2020 (The University of Tokyo Hokkaido Forest, 2025. Available online: https://www.uf.a.u-tokyo.ac.jp/hokuen/summary.eng.2025.pdf, accessed on 16 February 2026). Seasonal snow cover persists from mid-November through mid-May. The underlying bedrock is composed of rhyolite and dacite welded tuffs (Geological Survey of Japan 2003 [47]). The soils at the site are classified as dark forest soil [46]. Prior to disturbance, the site supported a mixed conifer–broadleaf forest typical of northern Japan. The dominant native tree species include *Abies sachalinensis*, *Picea jezoensis*, *Tilia japonica*, and *Betula ermanii*. The forest floor is largely occupied by dwarf bamboo, primarily *Sasa senanensis*, with some presence of *Sasa kurilensis*.

To facilitate rapid recovery of the affected forest under these climatic and site conditions, intensive silvicultural interventions were implemented immediately following the disturbance [46]. Salvage logging within the study area was conducted in August 1982, except in the designated control plots. Mechanical line scarification was carried out using two bulldozers in August 1983 (Komatsu D50A, 11 t, and D60A, 15 t) (Komatsu, Ishikawa, Japan). Scarified strips were established with a width of 5 m and spaced at 7 m intervals. Three scarification intensities were applied based on soil disturbance depth: 0 cm (removal of understory vegetation only), 10 cm (removal of the humus layer), and 20 cm (removal of surface mineral soil). Each treatment covered an area of 1.00 ha (100 m × 100 m). Reforestation treatments were also undertaken within the study site. Site preparation conducted in August 1983 involved bulldozer scarification forming 3 m wide strips with 4 m spacing between lines. In May 1984, four conifer species were planted across a total area of 20.82 ha, with two replicate plots established for each species: *Larix hybrid (L. gmelinii* var. *japonica* × *L. kaempferi*), *P. glehnii*, *P. jezoensis*, and *A. sachalinensis* [46].

The Mombetsu forest is encompassing approximately 18,199 ha in a hilly coastal landscape with elevations ranging from 40 to 400 m a.s.l. and classified as having a humid subarctic climate (Köppen Df), with a mean annual temperature of 6.1 °C and an average annual precipitation of about 836 mm. The forest operations are conducted by classifying the forest types, such as tall broadleaf-dominated mixed forests, short broadleaf-dominated mixed forests, mixed confier and broadleaf forests, tall conifer dominated mixed forests, short conifer-dominated mixed forests, and artifical plantations.

The study area comprises three sub-compartments in the Mombetsu forest: 114v (0.75 ha), 114w (0.75 ha), and 97g (0.75 ha). Sub-Compartments 114v and 114w are characterized by tall, broadleaf-dominated mixed forests with conifer plantations in the understory (hereafter referred to as Sub-Compartments 114v&w), whereas Sub-Compartment 97g consists of a short, broadleaf-dominated mixed forest (hereafter referred to as Sub-Compartment 97g). The derived elevation data indicate that Sub-Compartments 114v&w and 97g are located at elevations ranging from 134 to 215 m and 279 to 302 m a. s. l., respectively. These sub-compartments have been designated as silvicultural treatment areas. Partial cutting treatments, including 0%, 15%, and 35% thinning, were implemented to regulate stand structure and promote forest regeneration. These treatments were conducted in 1979 in Sub-Compartments 114v and 114w and in 2001 in Sub-Compartment 97g. The dominant tree species in sub-compartments include broadleaf species such as *Q. crispula*, *B. platyphylla*, *B. ermanii*, *B. maximowicziana*, *Acer pictum*, and *Kalopanax septemlobus*. *A. sachalinensis* occurs sparsely as a naturally mixed component in Sub-Compartment 97g, whereas it was planted in the understory of Sub-Compartments 114v and 114w following thinning conducted in 1979.

### 2.2. Data Collection

#### 2.2.1. Field Data

Field data were collected at the Sub-Compartment 97BC of the UTHF in June 2025. Trees with a DBH of 5 cm or greater were recorded at 1.3 m above ground level using a diameter tape in previously established sample plots. The plot sizes varied according to silvicultural treatments: approximately 10 × 10 m plots were used for scarification (9 plots) and control treatments (4 plots), while 15 × 15 m plots (16 plots) were used in plantation areas (Figure 1b).

In Sub-Compartments 114v&w of Mombetsu forest, field measurements were conducted in September and October 2025 within three existing experimental plots (approximately 20 × 20 m) per sub-compartment. Each plot represented a different cutting intensity (0%, 15%, and 35%) (Figure 1d), and all trees with DBH ≥ 5 cm were measured at 1.3 m above ground level using diameter tapes, following the same measurement procedure applied in Sub-Compartment 97BC of the UTHF. Prior to data collection, plot locations were confirmed using a dual-frequency global navigation satellite system (GNSS) receiver operating in real-time kinematic (RTK) mode (DG-PRO1RWS, Biz-Station Corp., Matsumoto City, Japan). For Sub-Compartment 97g, inventory data collected by Sumitomo Forestry Co., Ltd. in 2024 were used. This dataset comprised three approximately 20 × 20 m plots (Figure 1e), established using the same sampling design as in Sub-Compartments 114v&w, with one plot corresponding to each treatment level.

A single-variable volume equation based on diameter at breast height (DBH) was used to estimate the merchantable stem volume (V, m^3^) of individual trees across all study sub-compartments. The volume equation presented in Equation (1) was applied to conifer species, while Equations (2) and (3) were applied to broadleaf species for trees with DBH < 80 cm and DBH ≥ 80 cm, respectively [25], consistent with the applicable diameter ranges of the original volume equations developed for the University of Tokyo Hokkaido Forest. In Equations (1) and (2), d denotes DBH (cm), and log denotes the [base-10 (or natural)] logarithm, consistent with the original source. Carbon stock was estimated using Equation (4) based on the methodology described in the National Greenhouse Gas Inventory Report of Japan (2024). The mean and standard deviation (SD) of tree density, height, DBH, stand volume, and carbon stock for each silvicultural treatment are summarized in Table 1.

log *V* = −3.7789 + 2.4437 log *d* (1)

log *V* = −3.7844 + 2.4189 log *d* (2)

V = −8.62 + 0.190d(3)

(4) $$CST=\sum_{j}\left[(Vj\times Dj\times BEFj)\times (1+Rj)\times CF\right]$$

where:

V = merchantable stem volume (m^3^)
CST = carbon stock in living biomass (Mg C ha^−1^)
D = wood density (t–d.m. m^–3^)
BEF = biomass expansion factor (>20 years of age)
R = root-to-shoot ratio
CF = carbon fraction of dry biomass (MgC t–d.m.^–1^)
_j_ = species index

**Table 1:** Field-based descriptive statistics (mean ± SD) of tree density, height, DBH, stand volume, and carbon stock for each treated forest in the sub-compartments of the UTHF and Mombetsu Forest.

Sub-Compartment		Tree Density (Trees ha^−1^)	Height (m)	DBH (cm)	Stand Volume (m^3^ ha^−1^)	Carbon Stock (Mg C ha^−1^)
97BC	Scarification (*n* = 9)	1805 ± 994	14.83 ± 1.17	14.21 ± 5.97	189.02 ± 99.93	64.82 ± 33.46
	Plantation (*n* = 16)	1443 ± 610	15.14 ± 1.53	17.33 ± 2.94	293.48 ± 71.78	94.81 ± 21.64
	Control (*n* = 4)	1467 ± 437	13.49 ± 2.01	19.20 ± 4.28	499.21 ± 126.81	170.09 ± 20.94
114v&w	0% Cutting (*n* = 2)	1713 ± 40	11.96 ± 0.27	16.66 ± 0.00	422.62 ± 0.04	174.34 ± 0.93
	15% Cutting (*n* = 2)	1465 ± 338	11.22 ± 0.47	16.81 ± 1.93	327.26 ± 17.74	113.20 ± 1.86
	35% Cutting (*n* = 2)	1520 ± 270	12.36 ± 0.22	18.17 ± 0.85	421.04 ± 33.54	148.92 ± 29.94
97g	0% Cutting (*n* = 1)	2352	9.46	10.05	173.14	72.22
	15% Cutting (*n* = 1)	2348	8.21	9.36	123.13	50.50
	35% Cutting (*n* = 1)	2343	9.77	11.45	219.10	77.68

*n* = number of sample plots.

#### 2.2.2. Collection of UAV Imagery

**Figure 2:**
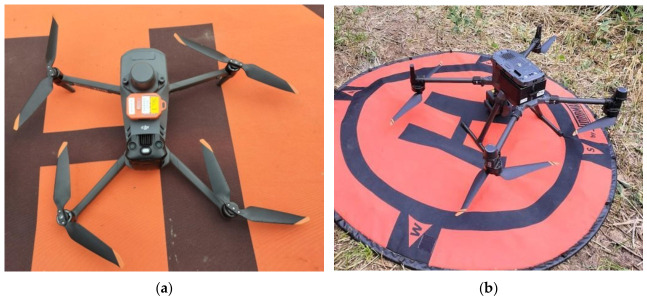
(**a**) DJI Mavic 3M UAV and (**b**) DJI Matrice 350 RTK UAV.

UAV imagery was collected over all the study sub-compartments using a DJI Mavic 3 Multispectral (Mavic 3M; DJI, Shenzhen, China) (Figure 2a). The platform is equipped with one RGB camera and four multispectral sensors capturing the green, red, red-edge, and near-infrared bands, enabling the derivation of vegetation indices used in this study. The UAV supports direct georeferencing through integration with a Differential RTK2 (D-RTK2) GNSS system, which can provide centimeter-level positioning accuracy for the acquired imagery [48].

Multispectral data acquisition was conducted during multiple flight campaigns to accommodate site accessibility and forest management schedules. UAV surveys were carried out at the Sub-Compartment 97BC of UTHF on 25 to 26 June 2025, while flights over Sub-Compartment 97g and other sub-compartments were conducted on 24 October 2024, and 30 September 2025, respectively. All surveys were conducted under suitable weather conditions using consistent flight parameters. Although the acquisition dates differed among study sites because of forest management schedules, the corresponding field measurements for each site were conducted within the same survey period so that the extracted spectral features were temporally consistent with the field reference data.

All flights were performed at a constant altitude of 80 m above ground level, with 90% forward overlap and 80% side overlap to ensure sufficient image redundancy for high-quality photogrammetric reconstruction. The multispectral and RGB cameras produced images with spatial resolutions of 1280 × 1024 pixels and 5280 × 3956 pixels, respectively. Identical flight parameters and sensor configurations were applied across all sub-compartments to maintain consistency in spatial resolution and data quality.

For the generation of the CHM at Sub-Compartment 97BC, an additional UAV-LiDAR survey was conducted on 23 June 2025, using a DJI Matrice 350 RTK UAV (UAV-LiDAR; DJI, Shenzhen, China) equipped with a Zenmuse L2 LiDAR sensor (Figure 2b). The UAV-LiDAR flights were carried out using the same flight parameters as those applied for the Mavic 3M surveys to ensure consistency in spatial coverage and data resolution.

### 2.3. UAV Image Preprocessing

The UAV imagery for all sub-compartments was processed using DJI Terra (version 5.1.0) (SZ DJI Technology Co., Ltd., Shenzhen, China), following the same workflow described in our previous study [48]. DJI Terra employs Structure from Motion (SfM) and Multi-View Stereo (MVS) techniques to reconstruct three-dimensional information from overlapping UAV images and also supports the processing of UAV-LiDAR point cloud data, including point cloud classification and the derivation of terrain-related products (e.g., digital elevation models) and surface models used for subsequent canopy analyses.

Radiometric calibration was performed using sensor-specific calibration parameters provided by the manufacturer, followed by reflectance normalization to ensure consistency across flight missions. Given the low-altitude UAV acquisition (~80 m above ground level) and short sensor-to-target distance, atmospheric effects were considered negligible in this study, consistent with common practice in UAV-based remote sensing of forest environments.

The processing workflow for UAV images acquired by the DJI Mavic 3M consisted of: (1) importing the images; (2) image alignment using SfM; (3) aerial triangulation and georeferencing; (4) the generation of sparse and dense point clouds using MVS; and (5) the production of RGB orthomosaic normalized vegetation index images (NDVI, GNDVI, and NDRE), and digital surface models (DSMs). All the generated products had GSDs of approximately 0.02 m. The integration of high-precision GNSS/D-RTK data supported the accurate georeferencing of the UAV-derived products, providing a reliable basis for subsequent analyses.

In addition, UAV-LiDAR data for Sub-Compartment 97BC were processed using the aforementioned DJI Terra, including point cloud classification into ground and non-ground returns, followed by the generation of a LiDAR-derived DSM and digital elevation model (DEM) to derive the CHM. In contrast, for the Mombetsu forest sub-compartments, DSMs and DEMs were generated using airborne LiDAR data acquired in August and September 2023 with a Cessna 208 Caravan I (JA889P) (Cessna Aircraft Company, Wichita, KS, USA) equipped primarily with a Leica CityMapper-2 system (Leica Geosystems AG, Heerbrugg, Switzerland) and MFC150 camera (Leica Geosystems AG, Heerbrugg, Switzerland). In areas lacking CityMapper-2 coverage, supplementary LiDAR and imagery data were acquired using a Leica TerrainMapper system integrated with an RCD30 camera (Leica Geosystems AG, Heerbrugg, Switzerland). In this study, the CHM for the Mombetsu forest was provided by the Mombetsu City Office, while the CHMs for Sub-Compartment 97BC were calculated in QGIS Desktop 3.40.11 as the difference between the DSM and DEM, and used in further analyses. The CHMs for the Mombetsu site, originally with a spatial resolution of 0.5 m, were nominally resampled to 0.02 m using nearest-neighbor interpolation solely to achieve spatial alignment with the corresponding orthophotos, near infrared (NIR) imagery, and spectral indices during feature extraction. This resampling was performed for geometric co-registration only and did not introduce additional structural information, improve the effective spatial resolution of the LiDAR-derived CHM, or alter the underlying canopy height measurements (ESRI Documentation, available online: https://doc.esri.com/en/arcgis-pro/latest/help/analysis/spatial-analyst/performing-analysis/cell-size-and-resampling-in-analysis.html, accessed on 10 July 2026). Consequently, the extracted structural features remained fundamentally constrained by the native 0.5 m LiDAR resolution.

All the spectral indices and structural metrics were derived systematically from the UAV multispectral imagery and LiDAR products, and all candidate features were retained as model inputs without manual feature selection. This approach ensures consistency and avoids subjective bias in feature engineering.

### 2.4. Data Analysis

**Figure 3:**
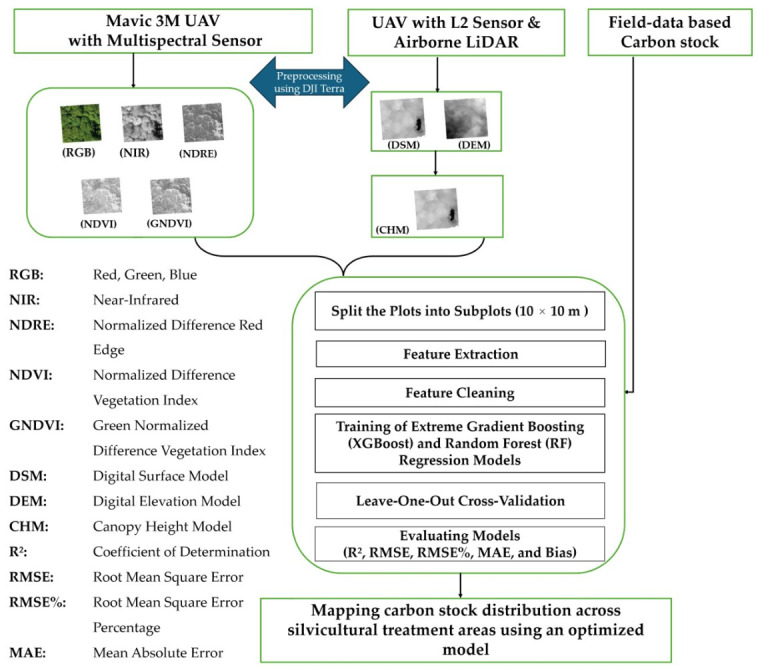
Overview workflow of carbon stock estimation using multi-sensor machine learning framework and wall-to-wall mapping using the optimized model.

Following the description in Section 2.1, the study area consisted of two geographically distinct regions: one compartment located in the UTHF in central Hokkaido, and two compartments located in the Mombetsu Forest in eastern Hokkaido. To examine the influence of regional variability on model performance and transferability, three training strategies were evaluated: (i) models trained using data from the UTHF site only, (ii) models trained using data from the Mombetsu Forest sites only, (iii) models trained using a combined dataset including all study sites, and (iv) the combined dataset was used for final model development and subsequent large-scale carbon stock mapping while site-specific models were analyzed to assess regional effects. An overview of the study’s workflow is presented in Figure 3.

#### 2.4.1. Machine Learning Models for Carbon Stock Estimation

The carbon stock of living biomass was estimated using two ensemble-based machine learning models: RF and XGBoost. Both models are non-parametric and capable of modeling complex nonlinear relationships while effectively handling multicollinearity and mixed predictor types, making them well suited for forest carbon stock estimation using remotely sensed and field-derived variables [35,36,40,49]. In addition, these ensemble learning algorithms have consistently demonstrated robust performance for remote sensing applications with relatively limited numbers of independent field plots. Therefore, they were considered more appropriate for the present study than data-intensive deep learning approaches, which generally require substantially larger training datasets [50].

The RF model constructs an ensemble of decision trees using a bootstrap sampling procedure, in which each tree is trained on a randomly resampled subset of the training data [51]. At each node of a tree, only a randomly selected subset of predictor variables is evaluated to determine the best division of the data. This process encourages diversity among trees and reduces similarity in their predictions. For regression tasks, final predictions are obtained by averaging the outputs of all trees. This ensemble strategy reduces variance, mitigates overfitting, enhances robustness to noise, and improves generalization performance. In addition, RF provides measures of variable importance, enabling the identification of key predictors contributing to carbon stock estimation [52].

The XGBoost model is an advanced gradient boosting framework that builds decision trees sequentially, with each new tree trained to minimize the residual errors of the preceding ensemble [35]. Unlike RF, which relies on independent trees, XGBoost optimizes model performance through an additive learning process guided by a differentiable loss function. The algorithm incorporates both L1 and L2 regularization terms (corresponding to Lasso and Ridge penalties, respectively) to control model complexity and mitigate overfitting (XGBoosting, available online: https://xgboosting.com/xgboost-regularization-techniques/, accessed on 2 February 2026). Furthermore, XGBoost employs efficient computational strategies, including weighted quantile sketching, tree pruning, and parallel processing, allowing it to handle large and high-dimensional datasets efficiently [53]. By explicitly modeling complex interactions among remote sensing-derived predictors, XGBoost often achieves higher predictive accuracy than RF in carbon stock estimation tasks [12,22,38].

To provide a transparent benchmark for evaluating the added value of the ensemble learning models, an MLR model was also implemented as a baseline. Unlike RF and XGBoost, MLR assumes a linear relationship between predictor variables and carbon stock and does not explicitly model nonlinear interactions. The baseline model was included solely for comparative purposes to assess the performance gains achieved by the nonlinear machine learning approaches.

#### 2.4.2. Training Machine Learning Models for Carbon Stock Estimation

##### Field-Based Carbon Stock Data

Field-based carbon stock measurements were obtained at the plot level and linked to spatial plot boundary polygons. Carbon stored in living biomass was recorded in megagrams per plot (Mg C plot^−1^), while plot areas were calculated from polygon geometries measured in the field using GNSS, as described in Section 2.2.1. To enable comparison among plots of differing sizes, carbon density was additionally expressed on an area-normalized basis (Mg C ha^−1^).

##### UAV- and LiDAR-Derived Predictor Variables for Carbon Stock Modeling

UAV-derived multispectral imagery and LiDAR-derived CHMs were used as predictor datasets for carbon stock estimation. The multispectral datasets included RGB orthomosaics, NIR imagery, and vegetation indices, including NDVI, GNDVI, and NDRE. Structural information was obtained from CHMs derived from both UAV-based and airborne LiDAR datasets. Multispectral data, such as NIR reflectance and vegetation indices, have been widely used for carbon stock estimation, as these spectral variables provide information on canopy density and leaf area, which are closely associated with aboveground biomass and carbon stock [21,54]. UAV-derived RGB imagery additionally provides additional information on canopy condition and horizontal structure [55,56,57]. Furthermore, CHM-derived structural metrics have demonstrated strong relationships with aboveground biomass and carbon stocks in previous studies [43,44,58,59].

To systematically evaluate the contribution of UAV-derived spectral information under controlled structural conditions, three raster configurations were prepared for model training: (i) a comprehensive dataset incorporating all available raster information, including multispectral imagery and CHM (RGB, NIR, vegetation indices, and CHM); (ii) a reduced dataset consisting of RGB imagery and CHM; and (iii) CHM only. All raster layers were georeferenced to a common coordinate system and spatially aligned with plot polygons. Raster values were extracted using polygon masking so that only pixels within plot boundaries contributed to feature computation.

##### Subplot Generation and Carbon Allocation

To better utilize the high spatial resolution of UAV-derived and LiDAR- derived datasets while maintaining compatibility with plot-based field measurements, each plot polygon was subdivided into regular 10 × 10 m square subplots. Subplots smaller than 5 m^2^ were excluded from analysis. Field-measured carbon stock was proportionally allocated to subplots according to their relative area within each plot using the average aggregated approach, due to the absence of subplot-level field carbon measurements [2,42]. This approach assumes a homogeneous carbon distribution within individual plots while allowing spatial variability to be represented through UAV- and LiDAR-derived features.

##### Feature Extraction

For each subplot, a set of handcrafted spectral, structural, and object-based canopy features were derived. When valid raster data were unavailable for a given subplot, feature extraction was performed at the plot level for that subplot only.

Spectral features were computed from NIR and vegetation index layers, including mean, median, maximum, and minimum values and standard deviation. From RGB imagery, band-wise mean, minimum, and maximum reflectance values were extracted for the red, green, and blue channels. Structural features were derived from the CHM after removing values below 0.2 m to suppress ground and understory influences. Height-related metrics included the mean, median, minimum, maximum, and percentile heights (10th–99th percentiles).

To further capture horizontal canopy organization, an object-based image analysis technique was applied [60,61]. Connected-component analysis of the filtered CHM enabled the delineation of canopy objects. For each object, crown-level geometric attributes were quantified, including crown area, defined as the planar polygon area (m^2^) [19]; circularity, calculated as 4π × Area/Perimeter^2^, representing the degree to which the crown shape approaches a perfect circle [62]; aspect ratio, defined as the ratio of the major to minor axis lengths of the crown [63]; and crown extent, representing the maximum horizontal crown diameter. These object-level descriptors provide additional structural information relevant to forest biomass and carbon stock variability. When multiple canopy objects were detected within a subplot, only the largest connected component was retained to represent the dominant canopy structure. The extracted multispectral and structural features are summarized in Table 2.

##### Feature Preprocessing and Selection

Prior to modeling, extracted features were preprocessed to improve robustness. Features with more than 70% missing values were discarded, remaining missing values were imputed using median values, near-constant predictors were removed, and highly correlated features were excluded using a Pearson correlation threshold of >0.95 [13,36,64]. Following preprocessing, RF variable importance rankings were used as a preliminary global feature-screening step to reduce the predictor set before model development [65,66]. This ranking was computed once using the available dataset prior to model training and was applied consistently to the RF, XGBoost, and MLR models to ensure a common basis for comparison. Initially, models were developed using the top 5, 10, and 15 ranked predictors. Additional feature subsets adjacent to the initial optimal configuration were subsequently evaluated to further refine predictor selection. For each candidate subset, the predictive performance was assessed using Leave-One-Plot-Out Cross-Validation (LOPO-CV), and the subset yielding the lowest RMSE was selected for the final analysis. The selected predictor subset was used consistently across the RF, XGBoost, and MLR models to ensure a fair comparison.

##### Model Training and Validation

Carbon stock estimation was conducted using RF, XGBoost, and MLR models. The MLR model served as a transparent baseline for comparison, whereas RF and XGBoost were the primary nonlinear machine learning models evaluated in this study. The model hyperparameters were optimized through a manual grid search combined with subplot-based Leave-One-Subplot-Out Cross-Validation (LOSO-CV), using RMSE as the optimization criterion.

For the RF model, the number of trees (n_estimators = 100–300), maximum tree depth (max_depth = unlimited or 5), and minimum number of samples per leaf (min_samples_leaf = 1–2) were tuned. For the XGBoost model, the learning rate (learning_rate = 0.05–0.15), maximum tree depth (max_depth = 1–3), and number of boosting iterations (n_estimators = 100–400) were optimized. The combination of hyperparameters yielding the lowest LOSO-CV RMSE was selected for each model. The MLR model was fitted using the same predictor variables and cross-validation framework as the RF and XGBoost models.

The final model performance for all three models (MLR, RF, and XGBoost) was evaluated using a LOPO-CV strategy to reduce the spatial dependence between training and testing data. No independent external validation dataset was available; therefore, all the reported performance metrics are based on cross-validation. In each iteration, all subplots belonging to one plot were withheld as an independent test unit, while subplots from the remaining plots were used for model training. Subplot-level predictions were aggregated to obtain plot-level carbon estimates, which were subsequently converted to per-hectare values.

All analyses were implemented in Python (3.12.3). The main libraries used included NumPy, pandas, GeoPandas, rasterio, scikit-learn, XGBoost, scikit-image, matplotlib, shapely, and joblib. Spatial data processing was performed using rasterio and GeoPandas, while machine learning models were implemented using scikit-learn (RF Regressor) and XGBoost (XGBRegressor). Image-based feature extraction was conducted using scikit-image, and geometric operations were handled using shapely. Random seed values were fixed across all experiments (random_state = 42) to ensure the reproducibility of model training and evaluation.

#### 2.4.3. Large-Scale Carbon Stock Prediction and Mapping

To extend plot-based carbon models to the landscape scale, the trained ensemble regression model was applied to UAV-derived raster datasets to generate spatially continuous carbon stock maps. Wall-to-wall prediction was conducted using a sliding-window approach, in which raster features were extracted from fixed-size windows corresponding to the subplot resolution used during model training (10 m × 10 m).

For each analysis window, the same spectral and structural features described in the previous section were extracted from co-registered UAV-derived products. To reduce the influence of non-canopy elements, CHM values below 0.5 m were excluded prior to feature extraction for large-scale analysis. The effective window area (m^2^) was also included as a predictor to ensure consistency with the subplot-based training framework.

As described previously in the Feature Preprocessing and Selection Section, feature vectors were cleaned and aligned to match the subset of predictor variables retained during model training, and missing values were imputed using median statistics derived from the training data. The optimized regression models were then applied to each window to predict the aboveground carbon density (Mg C ha^−1^). To reduce block-edge artifacts and stabilize predictions, overlapping window estimates were averaged at the pixel level, resulting in a continuous raster map of predicted carbon density.

The resulting carbon map was exported as a GeoTIFF raster and additionally converted to vector format for spatial analysis and visualization. The final wall-to-wall carbon map represents large-scale predictions generated using the optimized model trained on all available field plots. This approach enabled the spatially explicit estimation of forest carbon stocks across the entire study area, while maintaining consistency with the subplot-scale modeling framework and minimizing scale mismatch between field observations and UAV-derived predictors.

All the model training procedures and wall-to-wall predictions were conducted on a Dell Precision 5860 Tower (Dell Inc., Round Rock, TX, USA) workstation equipped with an Intel Xeon W5-2545 processor (Intel Corporation, Santa Clara, CA, USA) (12 cores, 24 threads; 3.5 GHz base frequency, up to 4.7 GHz) and 64 GB of RAM (2 × 32 GB).

#### 2.4.4. Evaluation Metrics

Model performance was evaluated using the coefficient of determination (R^2^), RMSE, relative RMSE (RMSE%), mean absolute error (MAE), and bias based on LOPO-CV at the field plot level. Carbon stocks were predicted at a 10 × 10 m spatial resolution and subsequently aggregated to the field plot level for validation. For comparability, error metrics (RMSE, RMSE%, MAE and bias), calculated at the plot level, were normalized by plot area and are expressed in Mg C ha^−1^.

To further evaluate the applicability of the optimized models across heterogeneous silvicultural treatment conditions, treatment-level analyses of field-based and predicted carbon stocks were additionally conducted. Given the limited number of field plots within individual treatment groups (*n* = 1–2 in Mombetsu sub-compartments), this assessment is intended to describe model behavior under different management conditions rather than to provide inferential or statistically robust treatment-level conclusions. Accordingly, results at the treatment level are presented solely for descriptive and exploratory purposes and should not be interpreted as statistical validation or evidence of treatment effects. The following formulas were used to compute the evaluation metrics (Equations (5)–(10)):

(5) $$R^{2}=1-\frac{\sum_{i=1}^{n}{\left(y_{i}-{\hat{y}}_{i}\right)}^{2}}{\sum_{i=1}^{n}{\left(y_{i}-{\bar{y}}_{i}\right)}^{2}}$$

(6) $$RMSE=\sqrt{\frac{1}{n}\sum_{i=1}^{n}{\left(y_{i}-{\hat{y}}_{i}\right)}^{2}}$$

(7) $$RMSE\%=\frac{RMSE}{{\bar{y}}_{i}}\times 100$$

(8) $$MAE=\frac{1}{n}\sum_{i=1}^{n}|y_{i}-{\hat{y}}_{i}|$$

(9) $$Bias=\frac{1}{n}\sum_{i=1}^{n}\left({\hat{y}}_{i}-y_{i}\right)$$

(10) $$Residual\;Percentage\;(\%)=\frac{\left({\hat{y}}_{i}-y_{i}\right)}{{\bar{y}}_{i}}\times 100$$

where

*y_i_* = Observed value for the *i*-th sample (e.g., field-measured carbon stock)
${\hat{y}}_{i}$ = Predicted value for the *i*-th sample (e.g., model-estimated carbon stock)
${\bar{y}}_{i}$ = Mean of observed values
n = Total number of observations (samples)
*i* = Index of observation (*i* = 1, 2, …, n)

## 3. Results

The results presented below are based on the final LOPO-CV evaluation. However, because feature screening and hyperparameter tuning were conducted prior to the final plot-level validation procedure, a limited degree of optimistic bias in the reported performance metrics cannot be entirely excluded.

### 3.1. Performance of Machine Learning Models Compared with a Multiple Linear Regression Baseline Using the Dataset of Sub-Compartment 97BC of the UTHF

**Figure 4:**
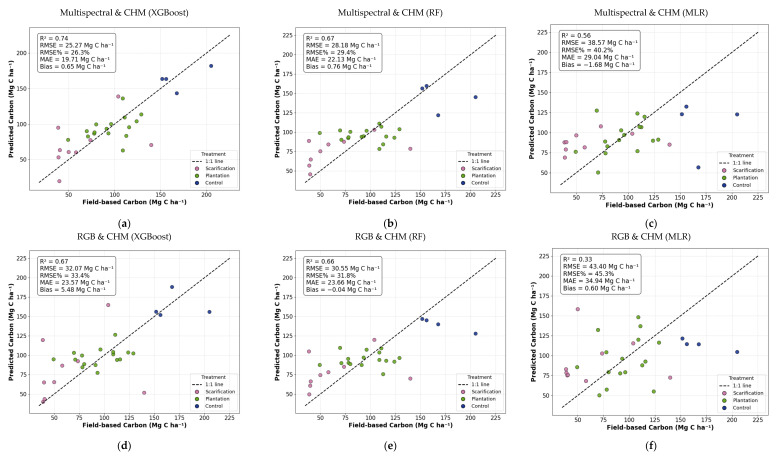

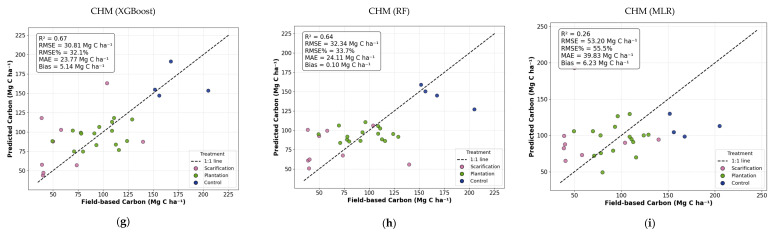
Field vs. Predicted carbon stock by XGBoost, RF and MLR models using the datasets from Sub-Compartment 97BC of the UTHF: (**a**–**c**) Multispectral & CHM dataset; (**d**–**f**) RGB & CHM dataset; and (**g**–**i**) CHM-only dataset.

Overall, the machine learning models outperformed the MLR baseline across all feature combinations, achieving higher R^2^ and lower prediction errors (Figure 4). Among the evaluated models, XGBoost consistently provided the best predictive performance, followed by RF, while MLR showed the lowest predictive accuracy. The best overall performance was achieved by the XGBoost model when CHM-derived metrics were combined with multispectral features. XGBoost attained the highest R^2^ value (0.74), along with the lowest RMSE (25.27), RMSE% (26.3%), and MAE (19.71). In comparison, the corresponding RF model exhibited weaker predictive performance, with a lower R^2^ (0.67) and higher RMSE (28.18), RMSE% (29.4%), and MAE (22.13). The MLR baseline produced the lowest predictive performance among the three models, with a lower R^2^ (0.56) and higher prediction errors (RMSE of 38.57, RMSE% of 40.2%, MAE of 20.04) than both RF and XGBoost.

When using the RGB and CHM feature set, XGBoost achieved an R^2^ of 0.67, with RMSE, RMSE%, and MAE values of 32.07, 33.4%, and 23.57, respectively. RF produced a comparable but slightly lower R^2^ (0.66) with similar error magnitudes. However, XGBoost showed a larger positive bias (5.48) relative to RF (−0.04), indicating a greater tendency toward overestimation under this feature configuration. When compared to the two machine learning models, the MLR baseline again showed lower predictive accuracy and higher error metrics (R^2^: 0.33; RMSE: 43.40; RMSE%; 45.3%; MAE: 34.94).

Model performance declined slightly for all models when only CHM-derived features were used. XGBoost achieved an R^2^ of 0.67, RMSE of 30.81, RMSE% of 32.1% and MAE of 23.77, while RF showed lower performance, with an R^2^ of 0.64, an RMSE of 32.34, and an RMSE% of 33.7%. The MLR baseline again produced the lowest R^2^ of 0.26 and the highest prediction errors among the evaluated models. Across all feature sets (Table 3), the inclusion of multispectral indices—particularly NIR, NDVI, and NDRE—in combination with CHM-derived crown metrics consistently improved model performance and reduced prediction error (Figure 4). These results emphasize the importance of integrating both structural and spectral information for accurate carbon stock estimation at this sub-compartment.

The field-versus-predicted relationships (Figure 4) revealed clear treatment-specific patterns across all feature configurations. Scarification plots, occupying the lower carbon range (approximately 40–80 Mg C ha^−1^), exhibited greater dispersion around the 1:1 line, particularly in models excluding multispectral indices. Several of these low-carbon plots were overestimated, most notably under the CHM-only configuration. Plantation plots, generally distributed within the intermediate carbon range (approximately 70–120 Mg C ha^−1^), showed tighter clustering and more stable predictions across feature sets. In contrast, control plots, representing the highest carbon stocks (≥150 Mg C ha^−1^), displayed a consistent tendency toward underestimation, especially in structural-only models. The inclusion of multispectral features reduced dispersion among scarification plots and improved the alignment of control plots with the 1:1 line, resulting in more balanced predictions across silvicultural treatments. These treatment-specific trends were generally consistent across the XGBoost, RF, and MLR models; however, the correspondence between predicted and observed carbon stocks was weaker for MLR across all feature configurations. Although incorporating multispectral features improved MLR performance, the gains were smaller than those observed for the machine learning models, and high-carbon control plots remained more frequently underestimated.

### 3.2. Performance of Machine Learning Models Compared with a Multiple Linear Regression Baseline Using the Dataset of Sub-Compartments 114v&w and 97g of Mombetsu Forest

**Figure 5:**
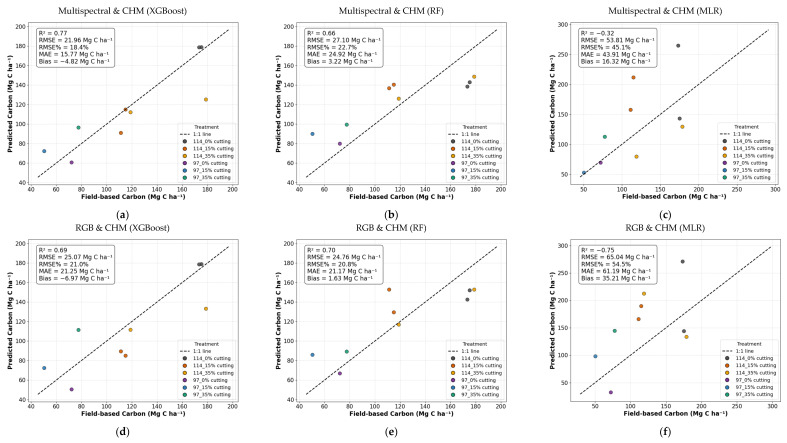

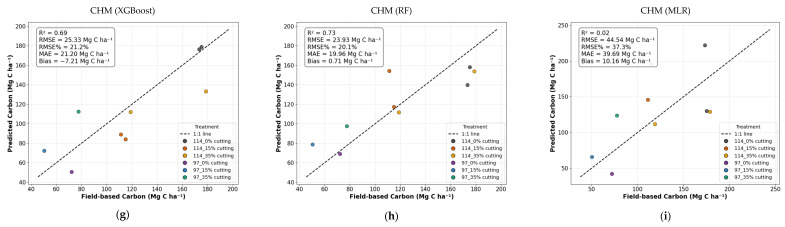
Field vs. Predicted carbon stock by XGBoost, RF and MLR models using the datasets from Sub-Compartments of Mombetsu Forest: (**a**–**c**) Multispectral & CHM dataset; (**d**–**f**) RGB & CHM dataset; and (**g**–**i**) CHM-only dataset.

Similar to the UTHF site, the MLR baseline exhibited lower predictive accuracy than the machine learning models for the Mombetsu dataset, with lower R^2^ values and higher prediction errors across the evaluated feature configurations (Figure 5). Among the evaluated models, XGBoost achieved its best predictive performance when CHM-derived metrics were combined with multispectral features, including RGB bands. Consistent improvement was observed with the inclusion of spectral features such as NDVI, NDRE, and GNDVI (Table 4). Under this configuration, XGBoost outperformed both RF and the MLR baseline, achieving a maximum R^2^ of 0.77, along with the lowest RMSE (21.96), RMSE% (18.4%), and MAE (15.77) (Figure 5a). In contrast, the RF model showed notably poorer performance, with an R^2^ of 0.66, RMSE of 27.10, RMSE% of 22.7%, and MAE of 24.92, indicating reduced explanatory power and higher prediction errors (Figure 5b). The MLR model yielded the lowest predictive performance, with an R^2^ of −0.32 and the highest error metrics (Figure 5c).

For the CHM and RGB feature set, the performance of all models declined. XGBoost achieved an R^2^ of 0.69, with RMSE, RMSE%, and MAE values of 25.07, 21.0%, and 21.25, respectively. RF produced a comparable R^2^ (0.70) and a marginally lower RMSE (24.76) and RMSE% (20.8%), with similar MAE values. However, XGBoost exhibited a larger negative bias (−6.98) than RF (1.63), indicating a stronger tendency toward underestimation under this configuration (Figure 5d,e). The MLR baseline again produced substantially lower R^2^ (−0.75), higher prediction errors, and larger bias than both machine learning models (Figure 5f).

When only CHM-derived features were used, RF marginally outperformed XGBoost in explanatory power, achieving the highest R^2^ (0.73) and lowest RMSE (23.93) and RMSE% (20.1%) among CHM-only models. XGBoost produced a slightly lower R^2^ (0.69) and higher error metrics. Nevertheless, both models showed reduced overall accuracy compared with configurations incorporating spectral information (Figure 5g,h). Nevertheless, both machine learning models continued to outperform the MLR baseline, which again exhibited the lowest R^2^ and the highest prediction errors among the evaluated models (Figure 5i).

The scatterplots in Figure 5 highlight treatment-specific differences in prediction performance across the 0%, 15%, and 35% cutting treatments within Sub-Compartments 114 v&w and 97g under different feature configurations. The Multispectral + CHM configuration generally showed the closest agreement with field-based carbon estimates, particularly for the 0% cutting treatment at higher carbon levels (approximately 170–180 Mg C ha^−1^), where observations were distributed near the 1:1 line with relatively low dispersion. Predictions for the 15% cutting treatment showed moderate variability within the intermediate carbon range (approximately 110–130 Mg C ha^−1^), while the 35% cutting treatment exhibited larger deviations from the reference line. The RGB + CHM configuration demonstrated intermediate performance, with slightly greater scatter and less consistent alignment compared with the Multispectral + CHM, especially for the 15% and 35% cutting treatments. In contrast, the CHM-only configuration showed the widest dispersion and greater deviations from the 1:1 line, indicating reduced ability to capture treatment-related variability using structural information alone. The relative performance of the three feature configurations was also reflected in the MLR model, with Multispectral + CHM providing the strongest results and CHM-only the weakest. However, MLR showed a reduced capacity to represent variation among the cutting treatments, resulting in lower overall predictive accuracy than the machine learning models.

### 3.3. Performance of Machine Learning Models Compared with a Multiple Linear Regression Baseline Using the Combined Dataset of Sub-Compartments of the UTHF and Mombetsu Forest

When combining the datasets from both sites, XGBoost generally outperformed both RF and MLR across the evaluated feature configurations. In particular, when CHM-derived structural metrics were integrated with multispectral information, XGBoost achieved the lowest prediction error.

**Figure 6:**
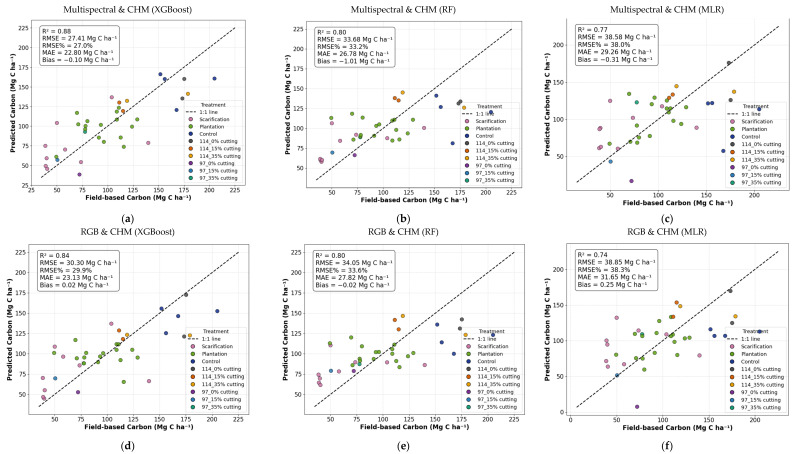

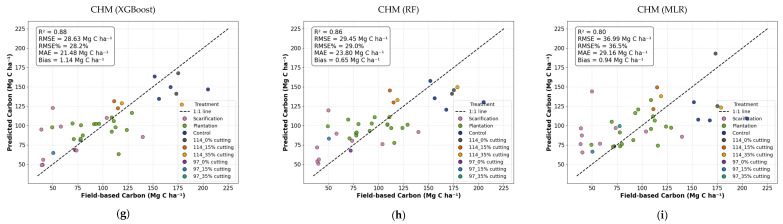
Field vs. Predicted carbon stock by XGBoost, RF and MLR models using the datasets from all sub-compartments: (**a**–**c**) Multispectral & CHM dataset; (**d**–**f**) RGB & CHM dataset; and (**g**–**i**) CHM-only dataset.

Under the combined Multispectral + CHM configuration, XGBoost achieved the highest predictive performance (R^2^ = 0.88; RMSE = 27.41 Mg C ha^−1^), followed by RF (R^2^ = 0.80; RMSE = 33.68 Mg C ha^−1^) and MLR (R^2^ = 0.77; RMSE = 38.58 Mg C ha^−1^). All three models exhibited negligible bias (−0.10, −1.01, and −0.31 Mg C ha^−1^ for XGBoost, RF, and MLR, respectively), indicating limited systematic prediction error despite differences in overall accuracy (Figure 6a–c).

A similar trend was observed for the RGB + CHM configuration, where XGBoost again achieved the highest predictive performance (R^2^ = 0.84; RMSE = 30.30 Mg C ha^−1^), followed by RF (R^2^ = 0.80; RMSE = 34.05 Mg C ha^−1^) and MLR (R^2^ = 0.74; RMSE = 38.85 Mg C ha^−1^). The bias remained negligible for all models (0.02, −0.02, and 0.25 Mg C ha^−1^ for XGBoost, RF, and MLR, respectively), although the MLR model produced the largest prediction errors (Figure 6d–f).

Using CHM-derived features alone, all three models retained relatively high explanatory power (R^2^ = 0.88, 0.86, and 0.80 for XGBoost, RF, and MLR, respectively), although the prediction errors were generally higher than those obtained using the Multispectral + CHM configuration. For XGBoost, incorporating multispectral information reduced the RMSE (28.63 to 27.41 Mg C ha^−1^) and bias (1.14 to −0.10 Mg C ha^−1^), while maintaining the same coefficient of determination (R^2^ = 0.88), despite a slight increase in MAE (21.48 to 22.80 Mg C ha^−1^). In contrast, the inclusion of multispectral features resulted in less consistent changes in the RF and MLR models. Although RF maintained comparable predictive performance across the three feature configurations, MLR derived comparatively limited benefit from the additional spectral information, with prediction errors remaining higher than those of the machine learning models. The CHM-only models exhibited positive bias, indicating a tendency to overestimate carbon stock when spectral information was not included (Figure 6g–i).

Feature selection results highlighted the dominant role of CHM-derived structural attributes, including maximum canopy height, crown extent, crown area, crown circularity, and height percentiles (e.g., chm_p20), which were consistently retained across feature sets. Spectral features—such as the NIR minimum, the NDRE mean and maximum, and selected RGB statistics—provided complementary contributions when combined with structural metrics (Table 5).

Scatterplots (Figure 6) demonstrated differences in prediction performance among feature configurations and treatment conditions. The Multispectral + CHM configuration generally showed the closest agreement with field-based carbon estimates, with many observations distributed near the 1:1 line across scarification, plantation, control, and thinning treatments, indicating comparatively stable performance. Moderate dispersion was observed in mid-range carbon stocks (approximately 80–130 Mg C ha^−1^), while some larger deviations remained in specific treatments, particularly the 97g 0% cutting plots. The RGB + CHM configuration showed intermediate performance, with slightly wider scatter and a tendency to underestimate several high-carbon plots, although it remained more stable than the CHM-only approach. In contrast, the CHM-only configuration exhibited the greatest dispersion and less consistent agreement with field-based estimates, especially in plantation, scarification, and 35% cutting plots. The MLR model showed trends consistent with those of the machine learning models, with Multispectral + CHM and RGB + CHM producing more reliable predictions than the CHM-only configuration. Although the linear model showed slightly lower agreement with field-based carbon estimates, the treatment-specific distributions were generally preserved across the combined dataset.

### 3.4. Carbon Stock Mapping Across Silvicultural Treated Forests in Sub-Compartments Using the Optimized XGBoost Model and Combined Dataset

**Figure A1:**
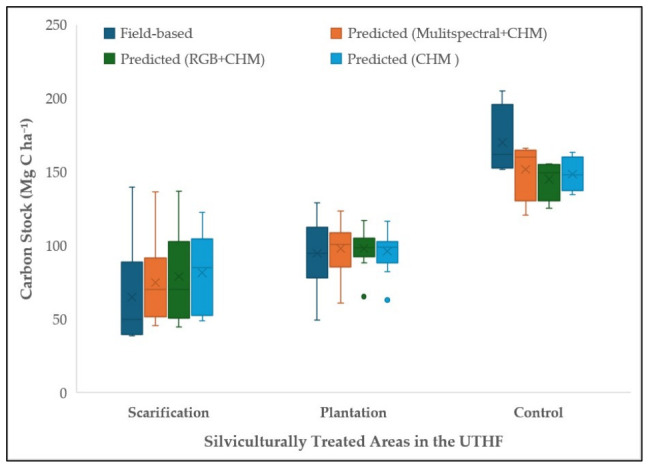
Descriptive comparison of field-based and model-predicted carbon stocks within silviculturally treated areas in Sub-Compartment 97BC under three feature configurations.

**Figure A2:**
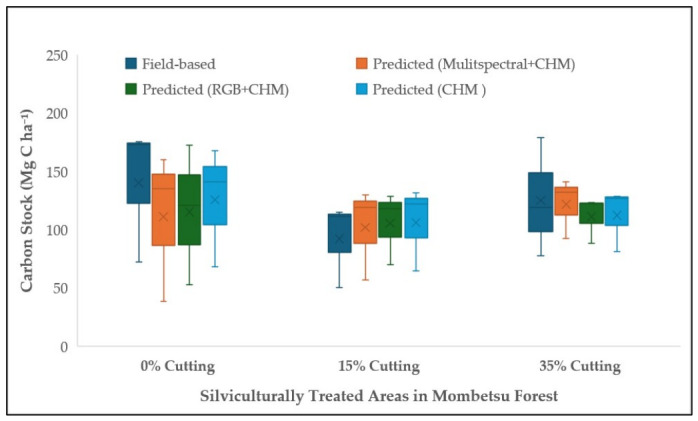
Descriptive comparison of field-based and model-predicted carbon stocks within silviculturally treated areas in Sub-Compartments 114v, 114w, and 97g under three feature configurations.

Treatment-level comparisons between field-based and plot-level predicted carbon stocks (Table 6) are presented as descriptive assessments of model behavior under different silvicultural conditions across all feature configurations. Under the Multispectral + CHM configuration, residual percentages were generally smaller in plantation and several cutting treatments within the 114v&w sub-compartment, whereas larger deviations were observed in scarification plots and the 0% cutting treatment in the 97g sub-compartment. Across all configurations, low-carbon treatment areas tended to be overestimated, while high-carbon stands were frequently underestimated, indicating a consistent range-dependent bias. The Multispectral + CHM configuration showed comparatively smaller residual variation across several treatment conditions and preserved greater separation among treatment-level carbon stocks relative to the RGB + CHM and CHM-only configurations. Plot-level field-based and predicted carbon stocks, together with residual percentages for each silvicultural treatment, are provided in Appendix A: Table A1, while Figure A1 and Figure A2 illustrate carbon stock variations across silvicultural treatment areas in the UTHF and Mombetsu Forest, respectively.

**Figure 7:**
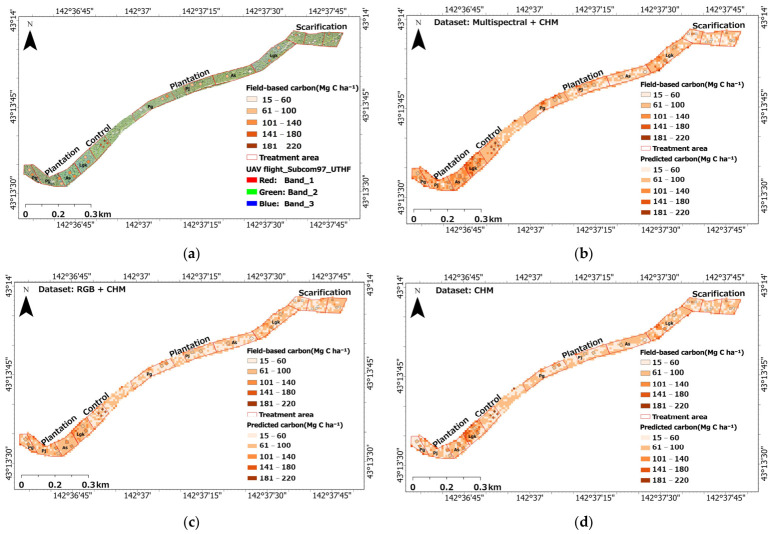
Wall-to-wall carbon stock predictions for Sub-Compartment 97BC derived from the optimized XGBoost model trained on combined datasets: (**a**) original orthophoto of Sub-compartment 97BC; (**b**) Multispectral + CHM-based prediction; (**c**) RGB + CHM-based prediction; and (**d**) CHM-only prediction.

**Figure 8:**
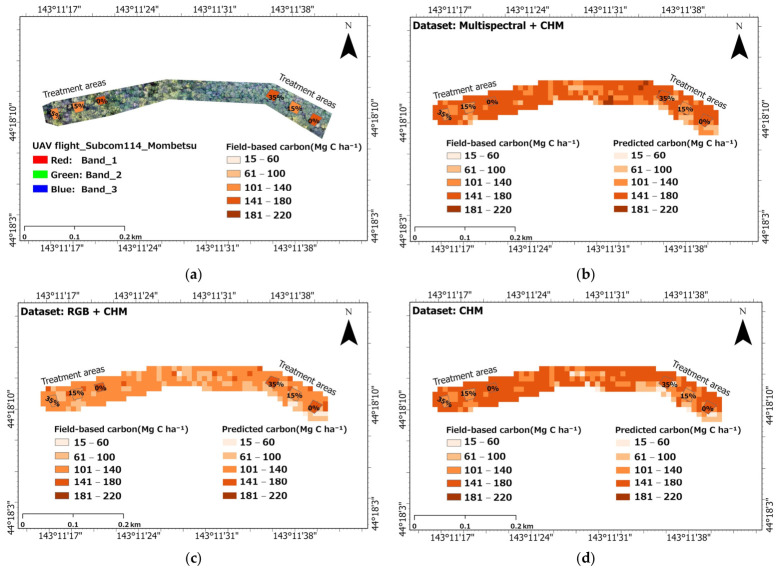
Wall-to-wall carbon stock predictions for Sub-Compartments v&w derived from the optimized XGBoost model trained on combined datasets: (**a**) original orthophoto of Sub-compartments v&w; (**b**) Multispectral + CHM-based prediction; (**c**) RGB + CHM-based prediction; and (**d**) CHM-only prediction.

**Figure 9:**
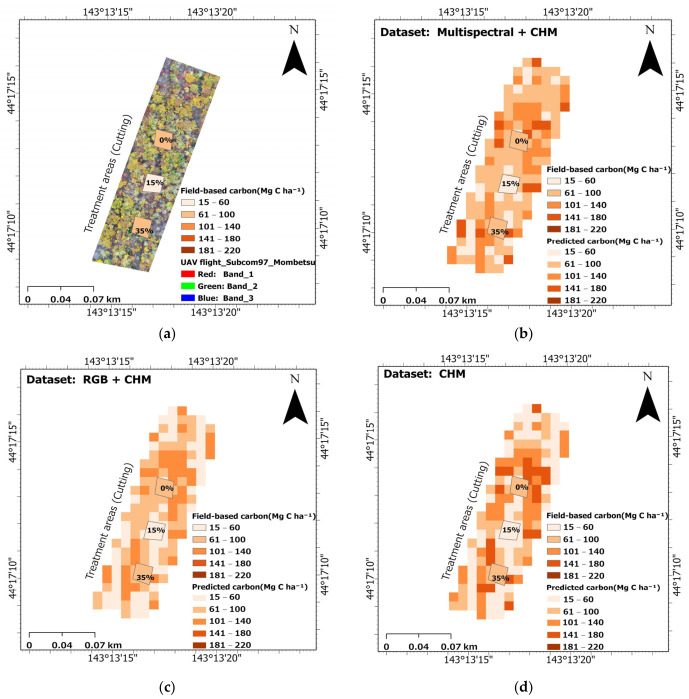
Wall-to-wall carbon stock predictions for Sub-Compartment 97g derived from the optimized XGBoost model trained on combined datasets: (**a**) original orthophoto of Sub-compartment 97g; (**b**) Multispectral + CHM-based prediction; (**c**) RGB + CHM-based prediction; and (**d**) CHM-only prediction.

The large-scale carbon stock distribution maps derived from the three feature configurations using the optimized XGBoost models are presented in Figure 7b–d, Figure 8b–d and Figure 9b–d. The mean field-based carbon stocks, calculated by averaging the sample plot data within each silviculturally treated forest, ranged from 50.51 to 174.34 Mg C ha^−1^, indicating substantial variability among management regimes. Table 7 presents a descriptive comparison between field-based mean carbon stocks and wall-to-wall predicted mean carbon stocks across silviculturally treated forests. Across all feature configurations, the mean residual percent revealed a consistent range-dependent bias.

The Multispectral + CHM configuration produced the most spatially coherent carbon distribution patterns and the smallest overall deviations from field-based means. For example, in the scarification treatment of 97BC, the field-based mean carbon stock of 64.82 Mg C ha^−1^ was predicted as 66.05 Mg C ha^−1^, corresponding to a residual of only 1.90%. Moderate residuals were observed in several mid-range treatment areas, whereas high-carbon areas were consistently underestimated, including the 15% cutting treatment in 114v&w (−15.63%) and the control stand in 97BC (−50.83%). Conversely, low-carbon treatment areas tended to be overestimated, particularly in 97g (15% cutting), where the residual reached 69.06%.

The RGB + CHM configuration exhibited substantial deviations across treatment regimes, particularly in high-carbon stands where underestimation was pronounced. For example, the control stand in 97BC showed a residual of −60.83%, while the plantation stand exhibited a residual of −21.97%. In contrast, several low- to moderate-carbon treatment areas showed overestimation, particularly in 97g, where the 15% cutting treatment exhibited a residual of 56.68%. Overall, the RGB + CHM configuration tended to underestimate carbon stocks in high-biomass stands while overestimating carbon stocks in lower-biomass treatment areas.

Under the CHM-only configuration, predicted carbon stocks were concentrated within the middle range, causing high- and low-carbon treatment areas to appear more similar and reducing the model’s ability to reflect differences in management intensity. High-carbon treatment areas, such as the control stand in 97BC and the 0% cutting treatment in 114v&w, were substantially underestimated, with residuals of −61.91% and −25.26%, respectively. In contrast, low-carbon stands, including all the cutting treatments in 97g, were consistently overestimated, with residuals ranging from 23.37% to 63.53%.

Overall, wall-to-wall predictions demonstrated a clear regression-to-the-mean pattern, characterized by the overestimation of low-carbon treatment areas and underestimation of high-carbon treatment areas. Spatially, the models predicted very low carbon values in areas with sparse or absent canopy cover, such as roads and open spaces within the study area. Among the three configurations, the Multispectral + CHM showed the closest agreement with field-based mean carbon stocks and better preserved relative differences among silvicultural treatments compared with the RGB + CHM and CHM-only configurations.

## 4. Discussion

### 4.1. Performance of Multi-Sensor Machine Learning Models for Carbon Stock Estimation

Across individual sites and the combined dataset, the results highlight the advantages of multi-sensor machine learning approaches that integrate UAV multispectral data with UAV- and airborne LiDAR-derived measurements for plot-level forest carbon stock estimation. In most modeling scenarios, XGBoost outperformed RF, particularly when CHM-derived structural metrics were integrated with multispectral information. This improved performance likely reflects XGBoost’s ability to capture nonlinear relationships and manage collinearity among high-dimensional multi-sensor-derived predictors, a finding consistent with previous UAV- and LiDAR-based carbon related biomass studies [12,22]. The baseline MLR model consistently produced lower predictive performance than the machine learning models, indicating that the relationships between multi-sensor-derived features and forest carbon stocks are not adequately represented by a simple linear model.

At Sub-Compartment 97BC of the UTHF, integrating multispectral indices (e.g., NIR, NDVI, and NDRE) with CHM-derived crown metrics substantially improved the predictive accuracy relative to structural-only models. Height-based metrics alone were insufficient to fully capture carbon stock variability across diverse silvicultural treatments. Crown extent and vegetation indices contributed strongly to model performance, whereas RGB features showed limited influence. The stronger contribution of spectral indices may be explained by the heterogeneous management conditions at this site, including scarification, plantation, and control plots, which introduce substantial variability in canopy cover, regeneration, and vegetation condition. In addition, summer (June) images were acquired under fully developed canopy conditions, which likely enhanced the sensitivity of spectral information to canopy vigor and leaf density compared with height-based metrics alone [67,68].

At sub-compartments of Mombetsu forest, XGBoost again achieved the highest predictive accuracy when structural and multispectral features were combined. Although RF marginally outperformed XGBoost under the CHM-only configuration, the overall accuracy remained lower than that of the multi-sensor models. Compared with Sub-Compartment 97BC, spectral features contributed less strongly, likely reflecting both the more structurally driven variability associated with different cutting intensities (0%, 15%, and 35%) and the timing of image acquisition during autumn, when partial senescence reduces the spectral sensitivity to the canopy condition. Nevertheless, spectral variables still provided meaningful information on vegetation condition [69,70].

Building on these site-specific analyses, we further evaluated models using a combined dataset from both study sites. The combined dataset encompassed a broader range of forest structures, species compositions, and carbon stock conditions, providing a more heterogeneous and operationally relevant dataset for model development. Although the combined-site model exhibited a slightly higher RMSE and MAE than the corresponding site-specific models, it maintained good predictive performance across this broader range of forest conditions, indicating the feasibility of developing a unified model for carbon stock estimation across heterogeneous managed forests. The higher R^2^ observed for the combined dataset should be interpreted in the context of the wider carbon stock gradient represented in the pooled data, rather than as a direct reduction in absolute prediction error. Feature importance analysis indicated that the combined model relied on a balanced set of structural and spectral predictors. Structural metrics (e.g., CHM-derived height and crown attributes) captured tree size and vertical complexity, which are primary drivers of biomass, while spectral variables provided complementary information on canopy density and vegetation condition.

Notably, NIR emerged as the most influential predictor in the combined dataset, although its importance varied between the individual sites. At the UTHF site, where the sample size was larger (*n* = 29), NIR was already identified as one of the most important predictors. Therefore, its high importance in the combined model may partly reflect the greater representation of UTHF samples compared with the Mombetsu site (*n* = 9). In addition, the increased variability in canopy structure and spectral conditions across sites likely strengthened the contribution of NIR-related predictors in capturing cross-site differences in canopy biomass. Furthermore, the RGB-derived variable red_mean was selected only in the combined dataset, suggesting greater spectral variability across sites, where differences in canopy condition or illumination may enhance the contribution of red reflectance in distinguishing canopy biomass as reported by previous studies [71,72,73].

Additionally, this improvement aligns with previous multi-sensor studies demonstrating that integrating LiDAR-derived structural metrics with spectral information—whether from satellite or hyperspectral platforms—enhances the accuracy and reduces the error of biomass and carbon stock estimation compared with single-sensor approaches [58,59,74]. LiDAR-derived structural features—including canopy height metrics and crown geometry descriptors—were consistently identified as dominant predictors, reflecting the critical role of structural information reported in similar fusion studies [58,59].

From the combined dataset, XGBoost models using Multispectral + CHM and CHM-only achieved high predictive performance (R^2^ = 0.88 for both), comparable to, or slightly higher than that in previous work in a UTHF sub-compartment using generalized linear mixed models with airborne LiDAR-derived features (R^2^ = 0.84) [25]. Furthermore, our findings are consistent with those of Karthigesu et al. (2024) [43], who reported R^2^ values of 0.37–0.93 for UAV RGB-derived spectral, textural, and structural features integrated with LiDAR-derived DEMs across two UTHF sub-compartments. Although direct comparisons across studies should be interpreted cautiously due to differences in modeling approaches, sampling design, and site conditions, these findings collectively reinforce the strong predictive value of integrating spectral and structural information.

While XGBoost generally achieved higher R^2^ and lower RMSE and MAE than RF and the baseline MLR model across most modeling scenarios, the consistently lower performance of MLR suggests that the relationships between structural and spectral predictors and forest carbon stocks are predominantly nonlinear. This finding highlights the advantage of machine learning algorithms, particularly XGBoost, in capturing complex interactions among multi-sensor-derived variables. However, XGBoost exhibited comparatively higher bias in several site-specific models, indicating a tendency toward systematic over- or underestimation. The only exception was the Multispectral + CHM from the combined dataset, where XGBoost achieved both superior accuracy and lower bias. The higher bias observed in some site-specific XGBoost models may reflect limited sample size and structural variability within individual sites, constraining generalization across the full carbon stock gradient. By contrast, the combined dataset increased the training variability and reduced systematic deviation, suggesting that while XGBoost more effectively minimized the overall prediction error, RF produced slightly more stable estimates under certain site-specific conditions.

Although the specific predictors selected varied among the UTHF site, Mombetsu forest site, and combined dataset, their functional roles were consistent. Structural metrics primarily explained the variability in carbon stock, while spectral features enhanced model stability across management gradients. Differences in feature importance likely reflect site-specific forest structure, species composition, management history, and the seasonal timing of UAV image acquisition. When data from both sites were combined, the models converged on a balanced set of structural and spectral predictors, indicating improved robustness and generalization across contrasting forest conditions.

### 4.2. Implications for Scalable, Wall-to-Wall Carbon Stock Mapping

The multi-sensor machine learning framework developed in this study has important implications for wall-to-wall carbon stock mapping in silviculturally treated forests. The combined dataset enabled the development of a single XGBoost model trained with a broader range of forest structures, species compositions, and management conditions. Within the study areas investigated, this model demonstrated the feasibility of generating spatially continuous carbon stock maps across heterogeneous managed forests.

Nevertheless, treatment-level analyses revealed substantial systematic deviations associated with carbon stock gradients and canopy structural conditions. Although plot-level predictions were generally closer to field measurements than wall-to-wall predictions, notable treatment-dependent residual variability remained evident, particularly in structurally extreme stands. For example, as shown in Table A1, Plot IDs 2, 6, and 26 exhibited relatively large overestimations of carbon stock. Plot 2 contained only four relatively large individuals (DBH 17.4–30.6 cm), resulting in a sparse stand structure. In contrast, Plots 6 and 26 were dominated by small-diameter trees, with most stems ranging from 5 to 12 cm DBH in Plot 6 and 6 to 15 cm DBH in Plot 26. These stand structures may have been insufficiently represented in the training dataset, limiting the model’s ability to accurately characterize their carbon stock and contributing to the observed overestimations. At the larger spatial scale, predictions frequently underestimated high-carbon stands and overestimated low-carbon stands, indicating persistent range-dependent bias under structurally heterogeneous forest conditions.

At the plot level, CHM-derived structural metrics alone explained a substantial proportion of carbon stock variability, confirming the value of LiDAR-based products as a foundation for large-area carbon mapping. However, for wall-to-wall mapping, CHM-only models exhibited substantial systematic bias, including overestimation in low-carbon plots and underestimation in high-carbon stands (Table 7), highlighting the limitations of relying solely on structural information. The RGB + CHM configuration improved performance relative to CHM-only models but remained less stable than the Multispectral + CHM configuration, particularly in low-carbon plots.

The integration of structural and spectral information improved the overall predictive stability and partially mitigated the range-dependent bias observed in structurally driven approaches [68], demonstrating that spectral features play a critical role in constraining model outputs across gradients of forest condition and management intensity. Nevertheless, deviations at structural extremes persisted, which may reflect differences in canopy structural heterogeneity and post-disturbance stand dynamics. Mature control stand, which achieved high carbon stocks due to the presence of a few large trees, was consistently underestimated, whereas the 15% cutting treatment in Sub-Compartment 97g, which had the lowest carbon stocks, was overestimated. Plantation and the cutting stands exhibited intermediate behavior, consistent with their more uniform canopy structure and reduced structural extremes. Mid-range carbon plots were predicted most reliably under the integrated configuration. At the plot scale, scarification plots characterized by sparse and discontinuous canopy structure tended to be overestimated at low carbon levels. However, when aggregated to a larger spatial scale, these localized overestimations were compensated by underestimations in denser plots, as well as by the averaging effect inherent in spatial aggregation [75]. As a result, the overall deviation from field-based estimates was reduced.

From an operational perspective, the multi-sensor machine learning framework presented in this study provides a practical pathway toward high-resolution carbon stock estimation at stand and sub-compartment scales, leveraging UAV-based multispectral imagery, LiDAR-derived structural products, and machine learning integration. The improved performance observed when combining data from multiple sites further suggests that such models are well suited for regional-scale carbon assessment, provided that sufficient variability in forest structure and management is included during model training.

Overall, these findings indicate that integrated UAV-based multispectral and LiDAR approaches can effectively bridge the gap between plot-level field measurements and spatially continuous carbon stock mapping. While minor systematic bias remains in structurally extreme conditions, the multi-sensor framework improves prediction consistency and reduces range-dependent error, supporting its application for operational forest carbon monitoring and management planning.

### 4.3. Uncertainty in Carbon Stock Estimation

#### 4.3.1. Data-Related Uncertainties

Uncertainty in forest carbon stock estimation arises primarily from data-related factors across multiple stages of the workflow, including field measurements, UAV image acquisition, and LiDAR-derived CHM generation.

Field reference carbon stocks were estimated using species-specific allometric equations rather than direct measurements. Consequently, uncertainties associated with the allometric models may propagate into the reference carbon estimates used for machine learning model development. In addition, standardized biomass expansion factors (BEF), root–shoot ratios, and carbon fraction values from Japan’s national greenhouse gas inventory were applied consistently across all study sites. Although these standardized parameters ensure methodological consistency, they may not fully capture variations in biomass allocation associated with stand structure, management history, or thinning intensity.

A key data-related limitation is observed in the Mombetsu site, where a temporal mismatch exists between airborne LiDAR acquisition and field/UAV campaigns. Specifically, CHMs were derived from airborne LiDAR data acquired in August–September 2023, whereas UAV multispectral imagery and field measurements were collected during 2024–2025. This temporal mismatch of approximately one to two years may introduce minor inconsistencies in canopy structure, particularly in actively managed stands where thinning, growth, and regeneration can alter canopy height over time.

In addition, UAV multispectral imagery was acquired on different dates across the study sites because of forest management schedules. Although UAV imagery and field measurements were collected within the same survey period for each site, seasonal differences in canopy phenology and illumination conditions among acquisition dates may have introduced additional variability into the spectral features, thereby contributing additional uncertainty to the developed models.

Furthermore, the Mombetsu CHM originally produced at a 0.5 m spatial resolution was resampled to 0.02 m to align with UAV-derived multispectral data. This resampling is purely for spatial co-registration and does not introduce additional structural information or increase the effective spatial resolution of the LiDAR-derived canopy surface. The resulting 0.02 m grid should therefore be interpreted as a geometrically aligned analysis grid rather than as a physically enhanced representation of canopy structure.

Additonally, the CHMs used in this study were derived from different LiDAR platforms, with UAV LiDAR used at the UTHF site and airborne LiDAR used at the Mombetsu site. Although both datasets were processed using standard normalization procedures and geometrically aligned for subsequent analyses, differences in point cloud density, scanning geometry, and acquisition characteristics may introduce systematic differences in the derived structural metrics. Consequently, CHM-derived predictors may not be directly comparable across sites, and residual cross-sensor biases may have contributed additional uncertainty to the machine learning models. Future studies should investigate cross-platform LiDAR harmonization and bias correction methods to improve structural comparability.

Finally, the number of field plots available for model development and evaluation was limited, and the distribution of plots among silvicultural treatment groups was uneven, with only one or two plots available for several Mombetsu sub-compartments. This reduces the statistical representativeness of treatment-level analyses, increases sensitivity to local stand variability, and limits the statistical confidence of comparisons. Therefore, treatment-wise results should be interpreted as descriptive rather than inferential. We also emphasize that increasing the number of independent field plots, together with subplot-level measurements, would improve the representativeness and reliability of the reference dataset, thereby providing a more robust foundation for future model development and validation.

#### 4.3.2. Model-Related Uncertainties

Additional uncertainty arises from the modeling framework itself. Although ensemble machine learning methods such as RF and XGBoost are effective for capturing nonlinear relationships between remotely sensed variables and forest carbon stocks, their predictive behavior can become less stable when trained on limited and imbalanced datasets. In particular, regression-to-the-mean effects may occur, leading to the systematic underestimation of high-carbon stands and overestimation of low-carbon stands. The underrepresentation of extreme carbon stock conditions in the training data likely contributed to these prediction biases.

Furthermore, no explicit bias correction or uncertainty-aware learning strategy was applied. Feature screening and hyperparameter optimization were performed prior to the final LOPO-CV evaluation. LOPO-CV was selected because the limited number of independent field plots made it important to maximize the amount of training data available in each iteration while preserving complete plot-level separation between the training and validation sets. Although predictive performance was assessed using independent plot-level validation, this workflow may introduce a limited degree of optimism in model selection because the feature selection and hyperparameter optimization procedures were not repeated independently within each cross-validation training fold. Future studies should adopt a fully nested cross-validation framework, in which feature selection and hyperparameter optimization are performed independently within each training fold, to provide a more rigorous assessment of predictive performance as larger field datasets become available.

The present study also did not include formal sensitivity analyses to evaluate the influence of uncertainties in spectral features, structural measurements, or field reference data on model predictions. Consequently, the robustness of the proposed framework to input perturbations, sensor noise, and varying environmental conditions was not explicitly quantified. Future studies should incorporate perturbation-based sensitivity analyses and uncertainty propagation methods to provide a more comprehensive assessment of model robustness.

Moreover, the present study relied on handcrafted spectral and structural features rather than automatically learned representations. Recent advances in deep learning, including convolutional neural networks, Vision Transformers, and Neural Radiance Field (NeRF)-based methods, have demonstrated strong potential for extracting informative spatial features from UAV imagery [31,32,33,76]. However, these approaches typically require substantially larger training datasets than were available in this study. Consequently, ensemble learning based on handcrafted features was considered more appropriate for the available field data. Future studies should investigate deep learning-based feature learning as larger datasets become available.

#### 4.3.3. Generalization and Transferability Limitations

Finally, limitations exist in terms of model generalization. The models were developed using data from two managed forest sites in Hokkaido, Japan, characterized by specific-species composition, stand structure, and silvicultural practices. Therefore, transferability to other forest ecosystems with different ecological conditions, disturbance histories, or management regimes has not been evaluated.

In addition, spatial extrapolation in wall-to-wall prediction introduces further uncertainty, particularly in areas where training data are sparse or where structural conditions fall outside the range of observed field measurements. Furthermore, the computational efficiency and scalability of the proposed framework for large-area operational applications were not formally evaluated, as the primary objective of this study was to evaluate the prediction performance across sensing configurations and machine learning models rather than computational efficiency. Accordingly, caution should be exercised when applying the proposed framework beyond the investigated forest conditions until further validation across broader geographic regions and forest types has been conducted.

## 5. Conclusions

This study developed and evaluated a multi-sensor machine learning framework for forest carbon stock estimation in structurally heterogeneous forests subjected to contrasting silvicultural treatments in central and eastern Hokkaido, Japan. Through systematic comparisons of CHM-only, RGB + CHM, and Multispectral + CHM sensing configurations, the results showed that incorporating multispectral information together with CHM-derived structural metrics generally improved prediction performance for the XGBoost model, while the magnitude of improvement varied across models and study sites. Compared with the baseline MLR model, the machine learning models consistently achieved higher predictive accuracy, suggesting that capturing nonlinear relationships between multi-sensor-derived structural and spectral predictors contributed to improved prediction accuracy. These findings also highlight the complementary roles of structural and spectral remote sensing information for carbon stock estimation in managed forests.

Among the evaluated machine learning models, XGBoost generally outperformed RF across the evaluated sensing configurations. The integration of multispectral and structural information produced the lowest prediction errors for XGBoost. The resulting wall-to-wall carbon stock maps illustrate the feasibility of producing spatially continuous carbon stock estimates using UAV-derived multispectral imagery and LiDAR-derived structural information within the investigated study areas.

Although the proposed framework showed promising performance within the investigated forest sites, further research is needed to improve its robustness and applicability across broader forest conditions. Future studies should incorporate formal sensitivity and uncertainty analyses, investigate approaches to reduce prediction bias, evaluate computational scalability for operational applications, and explore deep learning-based feature learning as larger training datasets become available. In addition, improving regional transferability through transfer learning and domain adaptation, together with integrating UAV-derived multispectral imagery, airborne LiDAR, and moderate-resolution satellite imagery, may further enhance large-scale forest carbon stock mapping.

## Figures and Tables

**Table 2:** Extracted features from each raster image.

Raster Image		Extracted Features
Multispectral	NIR, NDVI, GNDVI, NDRE	Mean, min, max, median, std
	RGB	Mean, min, max
Structural	CHM	Mean, min, max, median, std, p10, p20, p30, p_40, p_50, p_60, p_70, p_80, p_90, p_99, crown_area, crown_circularity, crown_aspect_ratio, crown_extent

**Table 3:** Selected features for each dataset identified using Random Forest variable importance, ranked in descending order of importance.

Dataset	Selected Features
Multispectral + CHM	‘chm_crown_area’, ‘nir_min’, ‘ndre_mean’, ‘ndvi_min’, ‘chm_crown_extent’ (5 features)
RGB + CHM	‘chm_crown_area’, ‘chm_crown_extent’, ‘rgb_red_mean’, ‘rgb_green_mean’, ‘rgb_green_max’, ‘chm_max’, ‘chm_p20’ (7 features)
CHM	‘chm_crown_area’, ‘chm_crown_extent’, ‘chm_min’, ‘chm_max’, ‘chm_p20’, ‘chm_crown_circularity’, ‘chm_std’, ‘chm_crown_aspect_ratio’, ‘chm_p10’ (9 features)

**Table 4:** Selected features for each dataset identified using Random Forest variable importance, ranked in descending order of importance.

Dataset	Selected Features
Multispectral + CHM	‘chm_min’, ‘chm_mean’, ‘chm_crown_circularity’, ‘gndvi_min’, ‘ndre_min’, ‘chm_max’, ‘chm_crown_aspect_ratio’, ‘gndvi_max’, ‘ndvi_min’, ‘rgb_green_mean’ (10 features)
RGB + CHM	‘chm_min’, ‘chm_crown_circularity’, ‘chm_mean’, ‘chm_max’, ‘rgb_red_min’, ‘rgb_green_min’ (6 features)
CHM	‘chm_min’, ‘chm_crown_circularity’, ‘chm_mean’ (3 features)

**Table 5:** Selected features for each dataset identified using Random Forest variable importance, ranked in descending order of importance.

Dataset	Selected Features
Multispectral + CHM	‘nir_min’, ‘chm_max’, ‘chm_crown_extent’, chm_crown_aspect_ratio’, ‘chm_min’, ‘ndre_max’, ‘chm_crown_area’, ‘ndre_mean’, ‘chm_crown_circularity’, ‘chm_p20’, ‘rgb_red_mean’, ‘rgb_green_max’ (12 features)
RGB + CHM	‘chm_max’, ‘chm_crown_extent’, ‘rgb_red_mean’, ‘chm_min’, ‘chm_crown_area’, ‘chm_crown_circularity’, ‘chm_crown_aspect_ratio’, ‘rgb_green_max’, ‘chm_p20’, ‘chm_std’ (10 features)
CHM	‘chm_max’, ‘chm_crown_extent’, ‘chm_crown_area’, ‘chm_min’, ‘chm_crown_circularity’, ‘chm_p20’ (6 features)

**Table 6:** Treatment-wise descriptive assessment of field-based and plot-level predicted carbon stocks across silvicultural treatments.

Sub-Compartment		Field-Based (Mg C ha^−1^)	Multispectral + CHM (Mg C ha^−1^)	Multispectral + CHM Residual %	RGB + CHM (Mg C ha^−1^)	RGB + CHM Residual %	CHM (Mg C ha^−1^)	CHM Residual %
97BC	Scarification	64.82	74.70	15	78.96	22	81.44	26
	Plantation	94.81	97.90	3	98.02	3	96.19	1
	Control	170.09	151.71	−11	144.96	−15	148.57	−13
114v&w	0% Cutting	174.34	147.56	−15	146.88	−16	154.32	−11
	15% Cutting	113.20	124.60	10	123.36	9	126.85	12
	35% Cutting	148.92	136.61	−8	122.94	−17	127.91	−14
97g	0% Cutting	72.21	38.38	−47	52.64	−27	68.27	−5
	15% Cutting	50.51	56.94	13	69.72	38	64.55	28
	35% Cutting	77.68	92.65	19	88.14	13	80.98	4

**Table 7:** Treatment-wise descriptive assessment of field-based and wall-to-wall predicted mean carbon stocks under different feature configurations.

Sub-Compartment		Field-Based (Mg C ha^−1^)	Multispectral + CHM (Mg C ha^−1^)	Multispectral + CHM Residual %	RGB + CHM (Mg C ha^−1^)	RGB + CHM Residual %	CHM (Mg C ha^−1^)	CHM Residual %
97BC	Scarification	64.82	66.05	2	68.02	5	68.26	5
	Plantation	94.81	85.79	−10	73.98	−22	72.89	−23
	Control	170.09	83.64	−51	66.63	−61	64.78	−62
114v&w	0% Cutting	174.34	147.09	−16	117.67	−33	130.31	−25
	15% Cutting	113.20	138.65	22	115.82	2	131.62	16
	35% Cutting	148.92	144.86	−3	117.37	−21	127.37	−14
97g	0% Cutting	72.21	100.65	39	92.66	28	98.26	36
	15% Cutting	50.51	85.39	69	79.14	57	82.60	64
	35% Cutting	77.68	99.39	28	87.06	12	95.83	23

## Data Availability

Due to the sensitive nature of the study sites and related forest management considerations, the UAV imagery and LiDAR datasets used in this research are not publicly available. However, they may be shared with interested researchers upon reasonable request to the corresponding author, provided that an appropriate justification for their intended use is submitted and any applicable data-sharing conditions are satisfied. The derived feature dataset and the model implementation code used in this study are also available from the corresponding author upon reasonable request.

## Appendix A

**Table A1:** Plot-level field-based and predicted carbon estimates for each silvicultural treatment.

Sub-Compartment		Plot-ID	Field-Based (Mg C ha^−1^)	Multispectral + CHM (Mg C ha^−1^)	Multispectral + CHM Residual %	RGB + CHM (Mg C ha^−1^)	RGB + CHM Residual %	CHM (Mg C ha^−1^)	CHM Residual %
97BC	Scarification	0	103.99	136.64	31	137.01	32	109.87	6
	Scarification	1	73.66	54.17	−26	85.54	16	67.76	−8
	Scarification	2	49.97	104.01	108	108.69	118	122.71	146
	Scarification	3	39.85	59.08	48	44.83	12	49.22	24
	Scarification	4	38.95	49.22	26	46.67	20	48.86	25
	Scarification	5	58.03	70.12	21	96.32	66	98.66	70
	Scarification	6	38.66	74.84	94	70.20	82	94.83	145
	Scarification	7	139.82	78.63	−44	66.30	−53	85.23	−39
	Scarification	8	40.46	45.58	13	55.07	36	55.78	38
	Plantation	9	71.00	102.28	44	94.29	33	82.45	16
	Plantation	10	108.97	108.29	−1	111.73	3	110.41	1
	Plantation	11	49.43	60.93	23	100.91	104	97.67	98
	Plantation	12	77.80	96.05	23	88.06	13	82.81	6
	Plantation	13	91.26	85.35	−6	89.52	−2	101.80	12
	Plantation	14	78.16	100.16	28	95.29	22	100.37	28
	Plantation	15	112.72	85.47	−24	92.08	−18	97.74	−13
	Plantation	16	93.25	101.49	9	96.55	4	102.10	9
	Control	17	204.94	160.51	−22	152.51	−26	146.70	−28
	Control	18	167.63	120.49	−28	146.37	−13	149.68	−11
	Control	19	151.71	165.98	9	155.64	3	163.42	8
	Control	20	156.09	159.87	2	125.32	−20	134.49	−14
	Plantation	21	79.95	106.31	33	100.96	26	87.27	9
	Plantation	22	110.93	123.37	11	111.56	1	105.78	−5
	Plantation	23	108.89	118.44	9	104.69	−4	92.05	−15
	Plantation	24	128.99	108.48	−16	95.21	−26	116.32	−10
	Plantation	25	115.77	73.67	−36	65.34	−44	63.02	−46
	Plantation	26	69.75	116.82	67	116.86	68	102.71	47
	Plantation	27	96.16	79.95	−17	100.52	5	102.28	6
	Plantation	28	123.91	99.27	−20	104.82	−15	94.27	−24
114v	0% Cutting	29	175.27	159.99	−9	172.58	−2	167.58	−4
	15% Cutting	30	111.34	129.95	17	128.63	16	131.42	18
	35% Cutting	31	178.86	141.07	−21	122.59	−31	127.07	−29
114w	0% Cutting	32	173.42	135.12	−22	121.18	−30	141.06	−19
	15% Cutting	33	115.06	119.24	4	118.08	3	122.27	6
	35% Cutting	34	118.98	132.15	11	123.29	4	128.75	8
97g	0% Cutting	35	72.21	38.38	−47	52.64	−27	68.27	−5
	15% Cutting	36	50.51	56.94	13	69.72	38	64.55	28
	35% Cutting	37	77.68	92.65	19	88.14	13	80.98	4
